# Supplementary material for: The effect of age on visuomotor learning processes
Source: PLoS One. 2020 Sep 14;15(9):e0239032. doi: 10.1371/journal.pone.0239032 (PMC7489529; doi:10.1371/journal.pone.0239032)
Supplement: Attachment — Submitted filename: Reply_to_reviewers.pdf [file pone.0239032.s002.pdf]

Pre-print feedback

REPLY: We received some feedback on our pre-print, and have made some smaller changes to the manuscript. Most notably however, there was a suggestion to show individual data in the figures. We agree this is good practice, so we extended all data figures.

Reviewer #1:

SUMMARY

The manuscript describes a study aimed to evaluate the effects of instruction and strategy use on visuomotor rotation ability during reaching movements and, importantly, how instruction and strategy use might interact with age. Their sample included older adults (mean age 20.9 years) and younger adults (mean age 70.0 years). All participants were adapted to a force field using an arm manipulandum. Some participants received instruction from the experimenter on how to counteract the force field and some participants did not. To assess implicit learning, the experimenters assessed aftereffects after adaptation. To assess sensory and non-sensory contributions, they measured each participant's ability to locate the place where their hand had been while moving the manipulandum after adaptation. The manipulandum was either moved by themselves (active) or by a robot (passive) and they were not able to see the hand being moved. Finally, to assess explicit learning, the experimenters used the between-participants instruction manipulation (instructed, not instructed) that occurred before adaptation and added a within-subjects strategy use manipulation (use strategy, don't use strategy). They asked participants to either use or not use a strategy while interacting with the manipulandum after adaptation. All measures were compared to a baseline that was the use of the arm manipulandum with no force field applied. During the first trials of adaptation blocks, instruction was more effective in younger adults than older adults during adaptation – younger adults who received instruction adapted faster than older adults who received instruction. After adaptation, aftereffects were similar between age groups, suggesting that implicit learning was similar in older and younger adults and unaffected by instruction. Active arm reaches with the manipulandum led to larger proprioceptive realignment (based on hand localization) than passive arm reaches, and this active/passive effect was greater in the older adults than in the younger adults. Instruction did not affect hand localization. Finally, they found an interaction between instruction and strategy after adaptation such that participants who received instruction from the experimenter in how to counteract the force field performed better than participants who received no instructions when they used that strategy compared to when they did not. Age did not interact with instruction and strategy use, suggesting that both age groups apply explicit instruction similarly after adaptation.

Taken together, their results suggest that older adults may have more difficulty implementing an instruction about how to adapt their arm movements when initially adapting to a new visuomotor mapping. This difficulty may be specific to the early stages of adaptation because they found that older adults apply strategy similar to younger adults after adaptation stabilizes and as the effects of adaptation begin to fade.

This is a generally well-written and methodologically sound manuscript. Most of my comments are minor. I have four overall comment followed by more specific comments.

REPLY: Before addressing individual comments, we would like to note that a lot of the feedback seems to stem from two misunderstandings, for which we apologize.

First and foremost, we did not clearly emphasize the main goal of our study. The main question is not about explicit learning in older compared to younger adults per se, although it is an important prerequisite. Rather, we're asking how hand localization signals (which we consider implicit) change in older and younger adults when these different age-groups rely on explicit (or implicit) adaptation to a different extent. For this, we need to be able to manipulate the extent of explicit learning, and ascertain that this works, without changing other aspects of the study (such as the rotation size). This means that we do spend a lot of time investigating explicit and implicit learning before being able to answer the main questions on hand localization signals. We understand this may be confusing and have now tried to explain the logic of the study better. As an example, here is part of the last paragraph in the Introduction (page 7, line 136-139):

...we use a 30° rotation which allows us to evoke explicit learning in half the participants by providing them with instructions [16,29]. This way we can directly assess the differences between adaptation with and without explicit contributions, particularly how this affects shifts in hand location estimates.

Second, there seems to be some doubt that explicit learning plays a role in visuomotor adaptation. Since this apparently goes against older, seminal work, this may sound puzzling to people outside the field. While our main audience is the motor learning community, where the existence of explicit contributions is mostly established, we do want our paper to be understandable by as many readers as possible. So we now briefly preface the part in the introduction on explicit learning with a few sentences to explain the

recent developments establishing the prevalence of this process (page 3, line 60-63):

Adaptation is traditionally thought to solely rely on implicit processes, which Stanley & Krakauer [19] suggest is a misunderstanding of seminal work, for example on patient H.M. [20]. While the idea is older [21], in the last decade or so, explicit processes have been established as a substantial contributor to motor learning, including visuomotor adaptation [4,22–25].

There are some other topics that weren't as clearly described as they should have been, and we've tried to address those as well, but solutions to these two issues should address the main concerns.

OVERALL COMMENTS

#1 As I understand it, the premise set forth by the authors is this: Older adults are slower at adapting to large rotations than younger adults. There is no difference between older and younger adults at adapting to small cursor rotations (although there is some evidence to the contrary). Together, the two findings suggest the hypothesis that older adults have preserved implicit processing with deficits in explicit processing. In support of this hypothesis, they note that there is no difference between older and younger adults in reach after-effects after adaptation, a process widely considered to be implicit.

To draw a parallel between large/small rotations and explicit/implicit processing, the authors need to provide more information: To what extent are larger cursor rotations truly driven by explicit processes compared to implicit processes? To what extent are smaller cursor rotations truly driven by implicit processes compared to explicit processes?

The authors address this missing information with statements, such as these:

Lines 57 – 48: "Adaptation requires both a cognitive, or explicit, component which tends to contribute to early stages of learning, as well as implicit processes that predominate in the later stages."

Lines 67 – 68: "Because larger rotations produce larger initial reaching errors than smaller rotations, they are more likely to require and evoke cognitive processes…".

They do not provide citations, however, or explanations of empirical work that would support the large/small rotations and explicit/implicit parallel.

This is very important, because the purpose of the study was to understand how age is related to explicit and implicit processes during reach adaptation.

REPLY: The larger contribution of explicit learning in larger rotations has been directly shown in a few studies (Werner et al., 2015; Bond and Taylor, 2015) including one of our own (Modchalingam et al., 2019). The exact extent of the explicit contribution has never been established, as far as we know. But, a guide could be work that claims that implicit adaptation can only account for about 15° of any rotation, so that the rest has to be explicit (Kim et al., 2018; Bond & Taylor, 2015). While this depends to some extent on how one interprets the data, our present study seems to confirm this. While we observe a general pattern in the literature that studies that find an effect of age tend to use larger rotations and studies that don’t find effects tend to use smaller rotations, this is not a strict rule. However, combined with work that establishes a link between age-related cognitive decline and motor adaptation deficits (Heuer, Hegele and colleagues, 2008; 2011; 2013; Noohi et al., 2016; Anguerra et al., 2010; Siedler et al., 2006) it seems reasonable to test if the effect of explicit adaptation on recalibration is different in older adults (e.g. Heuer & Hegele, 2008; 2011; 2013; Vandevoorde & Orban de Xivry, 2019; Noohi et al., 2016). As with many other (cognitive) tasks, differences between populations only show up with sufficient “difficulty”. We have tried to clarify those first sections of the Introduction, as our interpretation of the literature did serve to guide this study, and may be helpful in understanding the results.

#2 My second overall comment is related to #1. In the Introduction and Discussion, the authors indicated that age group differences between older and younger adults in adaptation are consistently found with large rotations (between 60 and 90 degrees), but not with smaller rotations. They hypothesize that this age group difference is due to a greater reliance upon explicit processes in older adults than in younger adults.

The stated purpose was to better understand how age is related to explicit and implicit processes during adaptation.

Why, then, did the authors chose a small rotation (30 degrees) for adaptation?

Additionally, much of the literature review in the Introduction as well as the literature referred to in the Discussion is about studies with large rotations. Would it be possible to bring in studies that used small rotations, given that this study used a small rotation?

REPLY: The main goal of the study was to investigate effects of age on hand localization signals in the context of explicit and implicit learning (see main reply). Since measures of changes in hand localization are often sensitive to the size of the perturbation, we wanted to evoke different amounts of explicit learning given the same rotation size, and that works best if the rotation is small enough to not evoke (much) explicit learning by itself. We used a 30° rotation, as our previous study (Modchalingam et al., 2019) showed that to be suitable for this purpose. We have now included this rationale for using a relatively small perturbation (page 7, line 133-139).

We have cited a fair number of studies with smaller rotations (Werner et al., 2015; Modchalingam et al., 2019; Bond & Taylor, 2015), including some that test age-related effects on visuomotor adaptation (Heuer & Hegele, 2008; Anguerra et al., 2013; Seidler et al., 2006) which is now extended with the suggestions by reviewer 3 (a small 30° rotation: Noohi et al., 2016 and a still modest rotation of 40°: Vandevoorde & Orban de Xivry, 2019). Our observation that larger rotations seem to evoke more age-related differences is shared by Heuer & Hegele (2008), who attribute this to increased "difficulty", and Buszard & Masters (2018) who observe that age-related effects are mixed for visuomotor adaptation but more clear for sequence learning which arguably requires more cognitive / explicit processes. Unfortunately, the literature comparing older and younger adults on adaptation to a smaller rotation seems rather limited. Also, while we would be happy to be shown more evidence going one way or another, we can not change the fact that this observation on our side did guide study design, and that it could provide an explanation for contradictory results in the literature as well as help put our results in a broader context.

#3 The Methods, Results, and the first paragraph of the Discussion were extremely clear. There are many places in the Introduction and Discussion, however, that were a bit more difficult to read. I think that some of the difficulty can be attributed to the presence of irrelevant details that made it hard to immediately see the most relevant point. Here is an example:

Lines 510 – 513: "Both of which have been shown to be unaffected by instruction or cognitive strategy [16,18] and are similar in magnitude whether the distortion is introduced gradually or abruptly (as demonstrated across different studies using the same setup; e.g., [46,57])."

The portion in italics is less relevant (maybe not at all relevant?) and, therefore, adds confusion to the sentence and paragraph. Please consider a revision of the Introduction and Discussion that removes these add-ons. This will make the reading of those sections much easier because the reader will not have to filter out these less relevant parts to see the take-home point of the sentence/paragraph.

REPLY: Thank you. We've tried to improve clarity by removing irrelevant details throughout the manuscript. Particularly the Discussion has been shortened a lot.

#4 The Abstract does not appear to capture the same take home of the study that is presented in the first paragraph of the Discussion. The Abstract seems to focus on the active/passive results while the majority of the manuscript seems to focus on the explicit instruction/strategy use results. Please spend some time making the take homes presented in the Abstract and Manuscript more cohesive with one another.

REPLY: Very good point. As stated above, the main goal of the study was not explained well in the Introduction and Discussion. While it was already best stated in the Abstract, we've clarified it further, and have rewritten large parts of the Introduction and Discussion to better represent the main questions and findings. This is further enhanced by feedback from the other reviewers.

ABSTRACT

Line 3: Although grammatically correct, it sounds strange to follow "adaptation of movements" with "is". Might be better to say, "Movement adaptation … is", or, "Adaptation of movement … is".

REPLY: In line with some of the other feedback, we have rewritten the Abstract from scratch, so this line is no longer present. We hope the content is now more clear as well.

Lines 28 – 30: The sentence starting with, "Following visuomotor adaptation" feels rather opaque. Could it be simplified/clarified?

REPLY: see previous reply.

INTRODUCTION

Line 35: "persistent changes" is a confusing word pair. It's clear what you mean after a few rereads, but it would be better if confusing word pairs like that were not in the first sentence. Maybe something more like, "Our brain has evolved to adapt our movements to the environment and our body. Our movements are able to adapt to changes online as well as changes that persist in time."

REPLY: Thanks for highlighting this. We've rewritten most of the paragraph to clarify it, so the sentence is no longer present, but we hope the content is much clearer now.

Lines 36 – 37: "Reach adaptation is based on both explicit and implicit processes." Please provide a citation for this statement. Please also add some text that provides some indication of what you mean by the words explicit and implicit.

REPLY: see main reply.

Line 44: "although not for [11]." Should include the example to which they are referring, e.g., "although not for prism adaptation [11]" or "although see [11] for an alternative finding".

REPLY: We have used the latter formulation now (page 3, line 49).

Line 47 should start a new paragraph.

Line 57 should start a new paragraph.

REPLY: We respectfully disagree. The material is all part of one, not overtly long rationale, with the latter sentences (after line 57, now 55) explaining part of the goals of the study within the context of earlier work (from the earlier lines). Obviously, we have not done the best possible job to connect all concepts and ideas here, and we have attempted to clarify the writing (pages 3-4: lines 44-58).

Lines 57 – 58: I hesitate a bit here: "Adaptation requires both a cognitive, or explicit, component which tends to contribute to early stages of learning, as well as implicit processes that predominate in the later stages." It is possible that it only feels more explicit during the initial portion of adaptation – there are several studies that suggest that visuomotor actions, especially ballistic actions like reaching, can be performed entirely implicitly, where implicitly means unconsciously, and are rarely, if ever, affected by explicit processes, where explicit means conscious (e.g., work of Milner and Goodale). It's possible that my hesitation here could be addressed by providing your definition of the terms "implicit" and "explicit".

REPLY: see main reply.

Relatedly, does the literature referenced here have any bearing on whether adaption is affected by a participant's explicitly stated strategy or whether the participant's explicitly stated strategy is, in fact, related to the experience of the adaptation effect?

REPLY: see main reply.

Lines 67 – 69: "Because larger rotations produce … to evoke cognitive processes…" The logic of this statement is not clear. Please clarify.

REPLY: People never move flawlessly, but in daily life movement errors are usually small and inconsequential, and luckily we do not have to think about how to correct for those errors: this happens automatically / implicitly. When we introduce larger errors (e.g. playing ping-pong in the wind, or in laboratory experiments), the errors do not disappear as quickly, or even fully, and some explicit/ cognitive effort must be made on every movement - presumably because implicit processes are not sufficient to counter these larger

perturbations (Kim et al., 2018). This has been documented in quite a few studies now (e.g.: Bond & Taylor, 2015; Werner et al., 2015; Modchalingam et al., 2019) and was unpacked in the two sentences after this one. We have now rewritten the sentence (page 4, lines 71-73):

While small perturbations may be compensated for entirely implicitly, perhaps up to ~15° of rotation [35], larger perturbations additionally recruit explicit mechanisms [17], resulting in cognitively accessible strategies [15,16].

We also removed the second unpacking sentence as it is now superfluous.

Lines 71 – 72: Missing citation for the sentence beginning with, "This is in contrast …".

REPLY: See above.

Lines 74 – 75: "… explicit and implicit processes appear to contribute to different aspects of adaptation performances." The literature review that precedes this statement does not support this statement well. Please clarify the preceding literature review so that this conclusion statement is well supported.

REPLY: In rewriting the Introduction this statement was removed.

METHODS

Line 175: missing period at end of sentence

REPLY: Thanks, it has been fixed.

Figure 2 is a bit confusing because it seems to suggest that the presence/absence of instruction on the perturbation was manipulated within-participants (… because it's easy for the reader to make the mistake of equating 'strategy' and 'instruction'). Is there a way to make the figure clearer? Maybe add asterisks that state that the "strategy" could have been given to them (i.e., instructed) or made up on their own (i.e., not instructed). Maybe adding some clarification on this point throughout the paper

would help, too. I initially made the mistake of equating 'strategy' with 'instruction', and it took me a while to figure that out!

REPLY: We have added a few lines describing the groups (i.e. as early in the Methods as seemed reasonable) to explain this a bit better (page 8, line 164-168):

The instructions were meant to result in a cognitively accessible strategy, although participants could also spontaneously develop a cognitive strategy. The difference between the instructed and non-instructed groups in the availability of a strategy was tested by asking people to reach with or without their strategy – whether or not this strategy was obtained from instructions or spontaneously developed.

How easy/difficult/possible is it for a participant to not use a strategy once they have practiced it? In other words, how plausible are the 'strategy' and 'no strategy' conditions in practice?

REPLY: Participants in this task simply had to try to move their unseen hand to the target as best they could, as was shown to work in Werner et al., 2015. They were instructed to reach as if they were doing an aligned training task. Of course, implicit / unconscious adaptation still causes reach deviations, and this shows up very clearly in our results as reach aftereffects, which are considered non-strategic / implicit. In the data it can be seen that this implicit component is very similar in all four groups which is consistent with both the notions that this is an automatic process and that it is capped at some level (Kim et al., 2018).

Why do the authors not consider the strategy that the participants made up on their own a sort of explicit instruction? They assume the participant's own strategy is solidified enough that the participant can either use it or not use it because this is part of the test design. It seems to me that the difference is "explicit strategy developed by another person" and "explicit strategy developed by oneself".

REPLY: We do allow for participants to come up and use their own strategy. Our previous work has shown this not to reach detectable levels in a 30° rotation (Modchalingam et al., 2019) in younger adults. The current data shows this to be similar in older adults (as expected): the non-instructed groups (older & younger) do not reach

differently when asked to use or not use their strategy, therefore, they do not have a cognitively accessible strategy. This then makes the proper analysis and interpretation of the localization shifts much more straightforward, which was the intended effect of choosing the 30° rotation - that we do still have to verify here of course.

Line 267: typo, “leaning” should be “learning”

REPLY: Typo fixed.

RESULTS

The results plots are lovely. It was a bit difficult for me to keep in my mind which measure they were depicting, despite the name of the measure being in the image. I would recommend adding a more explicit statement in the figure caption about how the measure was calculated or, if feasible, adding a schematic that depicts that calculation of the measure to the figure. Just a suggestion.

REPLY: We’ve added a short description of how each dependent variable was calculated (page 14, line 300-306).

The ordering of the subsection is not consistent with the ordering of the subsections in the Methods sections. “No Cursor Reaches” is described last in the Methods, but it is not last in the Results. The order in the Methods is best, because it is more coherent with the timeline of the experimental procedures. Please reorder the subsections so that they are in the same order in the Methods and Results sections.

REPLY: We’ve thought about this ordering, and agree that a chronological order matching the experiment might have been clearer. However, since the meaning of the results of the hand localization tasks only become clear in the context of the results of the no-cursor tasks, the ordering in the Results (and Introduction and Discussion) is meant to follow the underlying logic of the study (perhaps the order of the tasks in the experiment should have followed that as well, but we can not change this anymore). That is: first we show that the instructions evoked explicit learning (and to a highly similar extent in the two age groups), and then we can see if effects of age and explicit learning (if any) interact in shifts

of hand localization. Therefore, we believe that the order of the Results is the best order for understanding each of the Results in context. In order to avoid confusion, we have made sure the text in the Methods now also follows the logic of the study (page 11-12, line 247-299), and we have tried to clarify this further in numerous spots in both the Methods and the Results (e.g. page 8, line 170-172; page 14, line 323-325).

Lines 327 – 331: “For those aware of the cursor rotation, the corresponding no-cursor reach deviations when asked to reach with a strategy should be larger than those when asked not to use the strategy. And, for those who are not aware, there should be no difference between these two no-cursor reach tasks. We used this process dissociation procedure (PDP), to determine whether this measure of awareness varied with age.”

This is very concise, but it needs to be expanded a bit. It’s not clear to me how the effectiveness of their strategy is an indication of their awareness.

REPLY: This process dissocation procedure (PDP) is a previously described method to measure cognitively accessibly approaches to a task: in this case explicit adaptation. We briefly reiterate the method here, and now refer the reader to other papers using a PDP approach to assess explicit adaptation. Also, whatever strategy participants may have, it does not necessarily have to be effective, it just has to have a measurable effect on the reaching behaviour when the strategy is applied at will or not. In short, for solutions to any problem to be considered explicit, they should be under some conscious control; at the very least, one should be able to use the solution or not use it. To clarify the sentences quoted above, we now preface them with this explanation, and provide references to papers establishing PDP for explicit visuomotor rotation adaptation. (page 17-18, line 374-380)

This is particularly important given the interaction between instruction and strategy that is reported and the conclusion starting on line 340, “… the effect of instruction on awareness was equal for both age groups.” It is possible that participants were equally aware of the perturbation in all conditions and the interaction was because the experimenter-provided strategy (i.e., instructed condition) was simply more effective than the strategies that the participants came up with on their own (i.e., not instructed condition). The conclusion would, then, be, “… the effect of instruction on the effectiveness of the strategy was equal for both age groups.”

REPLY: In principle we agree that the line is not optimally worded. However, if non-instructed and instructed participants adapted equally explicitly, they should all be able to change their behavior at will. Both groups are adapting, but it is clear that the non-instructed groups did so fully implicitly – as expected (Modchalingam et al., 2019; Werner et al., 2015). We think part of the confusion comes from saying that people were "aware of the perturbation". In previous work this is meant as awareness of the exact nature of the perturbation (a rotation of the feedback of about 30 degrees) as well as the strategy to counter this perturbation (rotate reaches in the opposite direction). We are sure that some participants in the non-instructed groups noticed some change, but since their motor system implicitly adapted, it would have appeared to them as a transient change, and consequently they were mostly not aware of how they (continued to) countered this perturbation, nor did they figure out the exact nature of the perturbation. We wanted to stick with the terminology used in the rest of the paper as well, so we changed the line to: "This suggested that the effect of instruction on the availability of an explicit strategy was comparable for both age groups." (page 17, line 391-393)

The Results section moves between present and past tense often. Past tense is standard. I would recommend a quick read through to ensure that it is in past tense. Here are a few that I caught, but there are likely others:

Line 357: "interacts" should be "interacted" and "replicates" should be "replicated"
Line 359: "are" should be "were"
Line 362: "leads" should be "led"
Line 363: "varies" should be "varied"

REPLY: Done.

DISCUSSION

This initial paragraph is quite strong (starting line 397). It very clearly explains and synthesizes the results. At the beginning of this paragraph the authors say, "we find clear evidence for age-related deficits in "explicit" aspects of adaptation", but by the end they say, "we find some suggestion of age-related deficits in the use of an explicit strategy." These two statements appear to contradict one another a bit. Please clarify. I would suggest removing the word "clear".

REPLY: Agreed. The paragraph has been edited quite heavily with this and other feedback in mind, so that it does not contain this apparent contradiction anymore. (page 20, line 448-463)

Lines 407 – 409: The sentence starting with, "However", uses a double negative at the end, "… as to be non-detectable in older adults but not younger adults" and is, consequently, difficult to read/comprehend. Please reword.

REPLY: Yes, we have reworded the line (page 20, line 458-459):

In addition, while there is an added effect of efferent-based signals on hand localization shifts in younger adults, this is not detectable in older adults.

Lines 414 – 416: The sentence starting with, "Using …" has something wrong with it grammatically. Or, maybe it's just missing a comma? Please edit.

REPLY: Yes, however, this line is no longer in the manuscript as we have greatly shortened that section of the Discussion while clarifying it.

Line 421: "demonstrate" should be "demonstrated"

REPLY: We find this confusing as well, but the guidelines suggest to stick to the present tense for the Discussion.

Line 428 – 429: "This suggests that the ability or willingness to adopt a novel explicit strategy decreases with age."

Is it possible that the ability or willingness to adopt a novel explicit strategy—at any age—could have some dependence on the magnitude of the rotation? It seems that your results really only speak to explicit/implicit processes at small rotations because you did not test at large rotations. You vaguely mention this in line 430 with, "… under certain perturbations." Could you make it more explicit that the certain perturbation to which you are referring is 30 degrees?

REPLY: Since previous work has already shown that there are some adaptation deficits in older participants (although very often without looking at the speed of adaptation) and since smaller rotations would be easier to adapt to, it seems unlikely that this age-effect will disappear with larger rotations. In fact, we expect it to be larger. We have now rephrased the final sentences of that paragraph like this(page 21, line 471-476):

... we show that age-related differences in applying a cognitive strategy are limited to only the initial stages of learning (specifically the first set of 3 trials), albeit for a smaller rotation of 30°. Our findings seem to indicate that the ability or willingness to adopt a novel explicit strategy decreases with age, while the ability to apply a learned strategy is not affected. How this affects older participants' ability to adapt to larger rotations remains to be seen.

Lines 465 – 505: I don't think this section is needed. Consider removing or refocusing it on the results of the current study. As it is, it seems more like background information for a study correlating cognitive measures with adaptation. I can see how it is related, but it is not needed and doesn't add much to the discussion, in my opinion.

REPLY: This section is about cognitive mechanisms that could be involved in the broader concept of "explicit" adaptation. If explicit adaptation is affected by age, then it should be possible to also find age-related effects in these mechanisms. And deficits in each one of the mechanisms should also manifest as distinct deviations in the pattern of adaptation. One of these mechanisms provides a possible explanation for the small effect we find on initial benefits of instruction. We now make this more clear. But we also agree that most of the section was not needed, so we shortened it a great deal as well. (page 21-22, line 490-507)

Reviewer #2:

This manuscript tested the effects of instruction and strategy use on how well older and younger adults were able to compensate for a 30-degree visuomotor rotation during reach-training and then use this strategy afterwards when reaching without a cursor. Training-induced changes in proprioceptive and predicted estimates of the adapted hand in the two age groups were then compared. They found that

instruction benefitted older adults less than younger adults during initial training, but that older adults exhibited a similar pattern in reach aftereffects, suggesting that older adults' strategy use could be evoked during no-cursor reaches after enough training. They also found that implicit changes (proprioceptive recalibration) and reach aftereffects in older adults were greater than those in younger adults, independent of their awareness of the rotation, perhaps due to age declines in proprioceptive acuity. From these results, the authors suggest that the explicit contributions to motor learning decrease with age, whereas the implicit processes remain intact.

In my view, this is an interesting and well-written manuscript with clear and motivated hypotheses. I do, however, have a list of questions / general concerns:

• Of the 4 younger and 3 older adults who were removed due to task incompletion, how many were in the non-instructed vs. instructed groups?

REPLY: This is now described in the section on participants in the Methods (page 8-9, line 171-178):

We excluded 4 younger (all from the non-instructed group) and 3 older (1 non-instructed, 2 instructed) participants. One older participant didn't complete the experiment. All other excluded participants often "leaned on" the robot manipulandum during the 300 ms pre-reach hold period (see below) so that when the handle was released they made a sudden hand movement toward themselves and away from the targets. That is why, in these participants, reach direction relative to the target direction could not be determined for a lot of trials. The data of the excluded participants was never fully pre-processed and hence not statistically analyzed nor included in the online data set.

• Regarding the experimental procedure, how long was each session?

REPLY: We now mention this on page 9, line 198-199:

The procedure and set up are similar to our previous study [16], and could be completed in 90 minutes (excluding breaks and instructions).

• Page 21, line 296, "We find…" should be "We found…"

REPLY: Thanks, this has been fixed.

• Pages 26-27, starting on line 424, “Using the same instructions…”. This sentence doesn’t make sense; is it supposed to be two sentences? Also, should it be “we find that instructed younger participants compensate more”?

REPLY: This whole section has been rewritten, so the sentence is no longer there.

• Was the age difference in recalibration with age significantly larger in older vs young adults?

REPLY: We don’t fully understand the question. In our data, both the active and passive localization shifts were larger in older as compared to younger adults by about an equal amount. This can be most easily explained if the component that is shared between active and passive localization; proprioception, shifts more in the older adults than in the younger adults, while the update of predicted sensory consequences is the same in both age groups. In the mixed ANOVA on localization shifts, the main effect of age group is significant (apart from the expected main effect of localization type), so that it can be said that recalibration is significantly larger in older as compared to younger adults.

• Although the authors offer an explanation based on previous studies that cannot explain the age difference in proprioceptive calibration, they do not really go into detail about potential explanations that can. Can the authors speculate as to why implicit processes might be enhanced with aging? Could this be due to compensatory mechanisms with aging? What kinds of experimental procedures could future studies use to understand the factors that influence the amount of recalibration?

REPLY: Those are good questions, that we are interested in as well, but that we have no definitive answer to. While we do not see a decrease in proprioceptive acuity in this study, this is conceivably still an explanation. There may also be a cerebellar explanation in that predicted sensory consequences might be updated less in older adults, hence leading to increased shifts elsewhere. Additionally, the compensatory mechanism (decreased explicit learning leading to increased implicit learning) is a good explanation as well (e.g. Vandevoorde & Orban de Xivry, 2019). The latter is hard to confirm in our data, which might be a drawback of the smaller rotation. There could also be another explanation: a functional, increased reliance

on implicit adaptation, predicated on experience with a higher cost/benefit ratio of explicit solutions. I.e. increased effortless implicit adaptation (such as proprioceptive recalibration) may lead to decreased effortful explicit adaptation (up to a point) which reverses the direction of causality. In rewriting the Discussion, we have added a paragraph dealing with these possibilities (page 25-26, line 592-611). For now, the real answer remains unclear, and likely consists of a combination of effects.

• In the first sentence of the conclusion, “This study demonstrates that age-related decline leads…” Decline in what? Cognitive function?

REPLY: That was indeed unclear, however, we have rewritten most of the Conclusion so that the line is no longer there. We hope it is clearer now.

Reviewer #3:

The authors have investigated age differences in implicit and explicit processes of sensorimotor adaptation. They found little effects of age on adaptation, but older adults exhibited greater effects on felt hand position post practice.

Vandervoorde & Orban de Xivry (2019 Neurobio Aging) have already investigated age differences in implicit and explicit adaptation processes. Noohi et al. (2016 Neuropsychologia) also address age differences in strategy use. It is novel here that the authors are looking at age differences in the felt hand position post learning, but the Vandervoorde paper should be discussed in the introduction to help place the current study better in context. The authors do not well address why their findings might differ from those of previous studies- why did they find differences in explicit strategy use whereas Vandervoorde report implicit model recalibration declines with age?

REPLY: We now include these studies in several relevant sections in the Introduction and Discussion. However, in the Noohi et al. study, there was no effect of age on the visuomotor adaptation task itself (like in ours, and they use the same rotation size), only on the questionnaire. We have used the questionnaire ourselves but find the results unreliable as the phrasing may be ambiguous, and (in our

dataset) the results of the questionnaire didn't correspond to behavioural measures. While the Vandevoorde & Orban de Xivry paper is very interesting, they used a 40° rotation, which might have evoked more spontaneous, explicit adaptation, allowing for actual compensation of smaller explicit contributions by implicit processes. However, they ran a comparable adaptation task twice and only once find an age effect – consistent with our observation that age effects are unreliable in adaptation to smaller rotations. In their error-clamped feedback task, the older adults show larger implicit recalibration – potentially in line with the larger (implicit) hand localization shifts we find here. Here we have attempted to keep explicit and implicit adaptation largely the same in older and younger adults, so that we can fairly assess and compare changes in some of the underlying implicit processes, which may all be part of implicit model recalibration.

The only difference between younger and older adults we found in adaptation itself was in the first three trials of rotated training in the instructed groups. That is, in initially applying a strategy received through instruction. After training there was little to no difference between older and younger adults in the overall explicit and implicit reach adaptation.

How were the older adults screened for cognitive status? This is particularly important here given the focus on explicit instructions and cognitive strategies.

REPLY: We did not get ethics approval to use any of the usual tests, as they technically require a trained clinical psychologist on site to administer them. However, after consulting with a clinical neuropsychologist, we decided that since all our older participants were living independently (not in a home), were able to travel to campus, that they found their way on campus, and showed up for their appointments on time means that they are in good cognitive health. Some worked on campus, were ex-employees or were recruited through a lecture series for alumni of the university.

How many trials were omitted due to the visual inspection process described on page 15? Please provide some visual examples of these trajectories, as well as for the data of subjects that were excluded.

REPLY: Good point. The number of missing trials is now added (page 14, line 309-315).

The authors should be commended for the sample size tested, which is larger than some studies using this paradigm. Was a power analysis conducted, either a priori or post hoc?

REPLY: Sample size is in the normal range for work in our lab for these types of experiments (~20 per group). We did not do a power analysis, as the effects we were going to test (age-dependent modulation of hand localization shifts by explicit learning) have not been tested before as far as we know (except in one previous study from our lab, that used a different method), and we consulted a statistician at our institute who advised us that power analyses are usually not that informative, except for standardized tests, but not for experiments that will usually use tests that are different from previous work. Nevertheless, we have added some power analyses to the notebook, but only for follow-up t-tests since power analyses for ANOVAs are very hard to interpret. Lastly, we aim for our data to be publicly available, not only to make sure our conclusions are trustworthy, but also to generate data sets that are maximally useful to others – and the data could be used for any power analysis in the future.

The manuscript needs some editing for language and word choice throughout.

REPLY: We hope that this has been addressed by following the suggestions from all reviewers aimed at increasing clarity.
