# Supplementary material for: The effect of age on visuomotor learning processes
Source: PLoS One. 2020 Sep 14;15(9):e0239032. doi: 10.1371/journal.pone.0239032 (PMC7489529; doi:10.1371/journal.pone.0239032)
Supplement: Attachment — Submitted filename: PONE-D-19-19971_Review.docx [file pone.0239032.s001.docx]

This manuscript tested the effects of instruction and strategy use on how well older and younger adults were able to compensate for a 30-degree visuomotor rotation during reach-training and then use this strategy afterwards when reaching without a cursor. Training-induced changes in proprioceptive and predicted estimates of the adapted hand in the two age groups were then compared. They found that instruction benefitted older adults less than younger adults during initial training, but that older adults exhibited a similar pattern in reach aftereffects, suggesting that older adults’ strategy use could be evoked during no-cursor reaches after enough training. They also found that implicit changes (proprioceptive recalibration) and reach aftereffects in older adults were greater than those in younger adults, independent of their awareness of the rotation, perhaps due to age declines in proprioceptive acuity. From these results, the authors suggest that the explicit contributions to motor learning decrease with age, whereas the implicit processes remain intact.

In my view, this is an interesting and well-written manuscript with clear and motivated hypotheses. I do, however, have a list of questions / general concerns:

- Of the 4 younger and 3 older adults who were removed due to task incompletion, how many were in the non-instructed vs. instructed groups?
- Regarding the experimental procedure, how long was each session?
- Page 21, line 296, “We find…” should be “We found…”
- Pages 26-27, starting on line 424, “Using the same instructions…”. This sentence doesn’t make sense; is it supposed to be two sentences? Also, should it be “we find that instructed *younger* participants compensate more”?
- Was the age difference in recalibration with age *significantly* larger in older vs young adults?
- Although the authors offer an explanation based on previous studies that *cannot* explain the age difference in proprioceptive calibration, they do not really go into detail about potential explanations that *can*. Can the authors speculate as to why implicit processes might be enhanced with aging? Could this be due to compensatory mechanisms with aging? What kinds of experimental procedures could future studies use to understand the factors that influence the amount of recalibration?
- In the first sentence of the conclusion, “This study demonstrates that age-related decline leads…” Decline in what? Cognitive function?
